# Experiences of Health Care Professionals Working Extra Weekends to Reduce COVID-19–Related Surgical Backlog: Cross-sectional Study

**DOI:** 10.2196/40209

**Published:** 2022-12-06

**Authors:** Clyde Matava, Jeannette P So, Alomgir Hossain, Simon Kelley

**Affiliations:** 1 Department of Anesthesia and Pain Medicine The Hospital for Sick Children Toronto, ON Canada; 2 Perioperative Services The Hospital for Sick Children Toronto, ON Canada; 3 Clinical Research Services The Hospital for Sick Children Toronto, ON Canada; 4 Division of Orthopaedics The Hospital for Sick Children Toronto, ON Canada

**Keywords:** staff, wait-list, surgery, health care delivery, patient safety, quality improvement, patient satisfaction, COVID-19, practice redesign, burnout, preoperative, pediatric, perioperative, surgery, surgical staff, surgeon, healthcare, health care, staff perception, workforce, stress, work, occupational health, occupational safety, perception, workload, nurse, nursing, anesthesiologist, health care provider, health care professional, cross-sectional, online survey

## Abstract

**Background:**

During the quiescent periods of the COVID-19 pandemic in 2020, we implemented a weekend-scheduled pediatric surgery program to reduce COVID-19–related backlogs. Over 100 staff members from anesthesiologists to nurses, surgeons, and administrative and supporting personnel signed up to work extra weekends as part of a novel weekend elective pediatric surgery program to reduce COVID-19–related backlog: *Operating Room Ramp-Up After COVID-19 Lockdown Ends-Extra Lists* (ORRACLE-Xtra).

**Objective:**

In this study, we sought to evaluate staff perceptions and their level of satisfaction and experiences with working extra scheduled weekend elective surgical cases at the end of the 3-month pilot phase of ORRACLE-Xtra and identify key factors for participation.

**Methods:**

Following the pilot of ORRACLE-Xtra, all perioperative staff who worked at least 1 weekend list were invited to complete an online survey that was developed and tested prior to distribution. The survey collected information on the impact of working weekends on well-being, overall satisfaction, and likelihood of and preferences for working future weekend lists. Logistic regression was used to estimate the association of well-being with satisfaction and willingness to work future weekend lists.

**Results:**

A total of 82 out of 118 eligible staff responded to the survey for a response rate of 69%. Staff worked a median of 2 weekend lists (IQR 1-9). Of 82 staff members, 65 (79%) were satisfied or very satisfied with working the extra weekend elective lists, with surgeons and surgical trainees reporting the highest levels of satisfaction. Most respondents (72/82, 88%) would continue working weekend lists. A sense of accomplishment was associated with satisfaction with working on the weekend (odds ratio [OR] 19.97, 95% CI 1.79-222.63; *P*=.02) and willingness to participate in future weekend lists (OR 17.74, 95% CI 1.50-200.70; *P*=.02). Many (56/82, 68%) were willing to work weekend lists that included longer, more complex cases, which was associated with a sense of community (OR 0.12, 95% CI 0.02-0.63; *P*=.01).

**Conclusions:**

Staff participating in the first 3 months of the ORRACLE-Xtra program reported satisfaction with working weekends and a willingness to continue with the program, including doing longer, more complex cases. Institutions planning on implementing COVID-19 surgical backlog work may benefit from gathering key information from their staff.

## Introduction

The COVID-19 pandemic has had a significant impact on health care delivery. Several jurisdictions canceled nonurgent surgeries repeatedly as each wave imposed pressure on the health care infrastructure [1-6]. Surgical wait-lists increased due to the cancelations, causing further delays to accessing surgical care [4,6-11]. In children, the timing of surgery can affect growth, development, and long-term outcomes [12,13]. Various models of increasing operating room throughput have been proposed to manage the COVID-19–related backlogs [8,14-18]. These include the triage of surgical patients for acuity and disease progression, longer operating hours during weekdays, addition of weekend surgical lists, or a combination of these [3,7,8,15,16,19,20].

During the quiescent periods of the pandemic between January and April 2021, our institution implemented a novel program to mitigate the rapid increase in the surgical wait-list: *Operating Room Ramp-Up After COVID-19 Lockdown Ends-Extra Lists* (ORRACLE-Xtra). The ORRACLE-Xtra program scheduled weekend elective surgery lists of high-volume, low-acuity daycare procedures to reduce COVID-19–related backlog at our tertiary pediatric hospital [7]. Having not historically scheduled elective surgery on weekends, these weekend lists were in addition to the planned weekday activity. The program hinged on staff volunteering for extra shifts, and they were encouraged to request lists of interest or those that worked with their personal schedules. As this program launched during the peak of the second wave, it was possible that participation in ORRACLE-Xtra would have a negative impact on staff due to the increased workload. To our knowledge, the impact of COVID-19 surgical backlog recovery work on staff has not been previously reported, particularly work performed during the pandemic. This information would be useful in the planning of COVID-19 surgical recovery activities, which depend greatly on human resources.

Our aims for this cross-sectional survey study were to (1) determine staff perceptions of, experiences with, and level of satisfaction with working extra scheduled weekend elective surgical lists in the 3-month pilot phase of ORRACLE-Xtra; (2) assess the likelihood of staff continuing to support our weekend program; and (3) identify factors that will maximize engagement in the weekend elective surgery program in the longer term.

## Methods

### Study Design and Participants

The study setting was the perioperative department at The Hospital for Sick Children in Toronto, ON, an academic pediatric hospital that serves children aged 0 to 18 years. We surveyed all perioperative staff who participated in the pilot phase of ORRACLE-Xtra. Based on a total of 118 eligible staff, we needed a minimum of 95 respondents to achieve a margin of error of 5% with a 95% CI. We followed the Consensus-Based Checklist for Reporting of Survey Studies (CROSS) guidelines for reporting surveys [21].

Staff received an email invitation to complete the online survey at the end of the 3-month pilot phase, with a reminder sent one week later. The survey remained open during April 2021. All responses were anonymous, and consent was implied by participation in the survey.

### Questionnaire

The ORRACLE-Xtra steering committee, which comprised representatives from staff groups and included nursing (preoperative, intraoperative, and postoperative), administrative support, technical support, and physicians (anesthesiologists and surgeons), developed the questionnaire. The survey collected information on overall satisfaction with working weekend elective lists, impact of working weekends on aspects of well-being (job satisfaction; sense of achievement, accomplishment, and community; burnout; career development possibilities; and increased workload), and likelihood of and preferences for working future weekend lists (Multimedia Appendix 1).

The survey included 12 questions. Questions 1 to 3 included information regarding each respondent’s role, department, surgical specialty lists they had worked on, and number of lists worked. Questions 5 to 12 ascertained the level of satisfaction with weekend lists, perceptions on well-being, and the respondents’ preferences for future weekend lists.

We tested the questionnaire according to previously published guidelines and methodology [21-25]. The process included internal piloting for clarity, flow, and timing using a convenience sample of 3 members of the ORRACLE-Xtra steering committee. No significant changes were needed following their feedback.

### Statistical Analysis

Survey responses were collected and managed using REDCap (Vanderbilt University), a secure online electronic data capture tool [26,27]. Data were summarized as frequencies or percentages for categorical variables. Univariate analyses were conducted to explore the association between covariates and outcome variables to identify the covariates to include in the final multivariable models. Logistic regression was used to evaluate the association of well-being with satisfaction with working weekend elective lists and willingness to work future weekend lists or future weekend lists with longer or more complex operative cases, adjusting for confounders and interactions. We dichotomized satisfaction with working on weekend elective lists as “yes” if respondents were “very satisfied” or “satisfied” (vs “neither satisfied nor dissatisfied,” “dissatisfied,” or “very dissatisfied”). We considered respondents willing to participate in future weekend lists and willing to sign up for weekend lists with longer or more complex cases if they reported “definitely,” “probably,” or “possibly” versus “probably not” or “definitely not.” We included covariates in the models for staff role (categorical); number of weekend shifts worked (continuous); each of the 5 surgical services (binary); sense of accomplishment, community, and well-being; burnout; career development possibilities; increased workload; and job satisfaction (binary: “a great deal,” “quite a bit,” or “somewhat” vs “very little” or “not at all”) (Multimedia Appendix 2). If staff worked more than 1 type of service list during the pilot, we classified them under the first surgical service. We reported results as odds ratios (ORs) and CIs, and a 2-tailed *P* value ≤.05 was considered statistically significant. Statistical analyses were performed using SAS (version 9.4; SAS Institute).

### Ethics Approval

This study was approved by the Quality Improvement Committee at The Hospital for Sick Children (QIP-2021-01-08).

## Results

### Demographics

Of 118 eligible staff, 82 (69%) responded to the survey. Table 1 outlines the demographics of respondents, including anesthesiologists, nurses, service attendants, surgeons, and surgical trainees. Staff worked a median of 2 weekend lists (IQR 1-9) with a cumulative total of 230 weekend lists.

**Table 1:** Characteristics of respondents (N=82).

Characteristic			Anesthesiologist		Nurse		POCU^a^ attendant		Surgeon		Surgical trainee
Respondents, n (%)			26 (32)		34 (41)		1 (1)		17 (21)		4 (5)
Weekend lists worked, median (IQR)			2 (1-2)		3.5 (2-6)		3 (3-3)		2 (1-4)		3.5 (3-4)
**Clinical area^b^, n**											
	Dentistry (n=15)	7		4		1		3		—	
	Ophthalmology (n=20)	8		5		1		6		—	
	Orthopedics (n=3)	3		—^c^		—		—		—	
	Otolaryngology (n=9)	4		2		—		3		—	
	Preanesthesia clinic (n=1)	1		0		—		—		—	
	Perianesthesia nursing (n=17)	0		17		—		—		—	
	Intraoperative nursing (n=2)	0		2		—		—		—	
	Plastic surgery (n=17)	5		8		—		2		2	
	Urology (n=14)	4		5		—		3		2	

^a^POCU: perioperative care unit.

^b^Some staff worked in more than 1 clinical area.

^c^Not applicable.

### Satisfaction With Working Weekends

**Figure 1:**
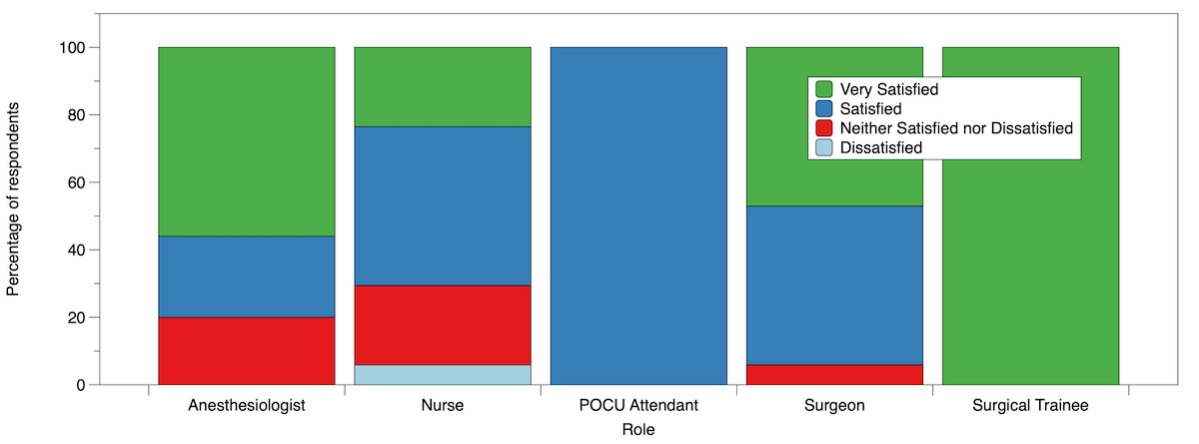
Staff satisfaction with working weekend elective lists. POCU: perioperative care unit.

Of 82 staff members, 65 (79%) were satisfied or very satisfied with working the extra weekend elective lists (Figure 1 and Multimedia Appendix 3). Surgeons and surgical trainees reported the highest levels of satisfaction. Only 2 (2%) out of 82 respondents expressed dissatisfaction with working the ORRACLE-Xtra weekends, and none reported being very dissatisfied.

#### Perceptions of Working Weekend Elective Lists

Most respondents perceived working the weekend elective surgery lists to have a positive impact on their sense of accomplishment and community, as well as overall job satisfaction. There were no statistically significant differences in self-reported career development, well-being, and burnout for staff who were satisfied with their weekend surgery experience. Surgeons and nurses were more likely to experience a sense of accomplishment from working the weekend lists while also being more likely to report that the work contributed to their sense of burnout.

**Figure 2:**
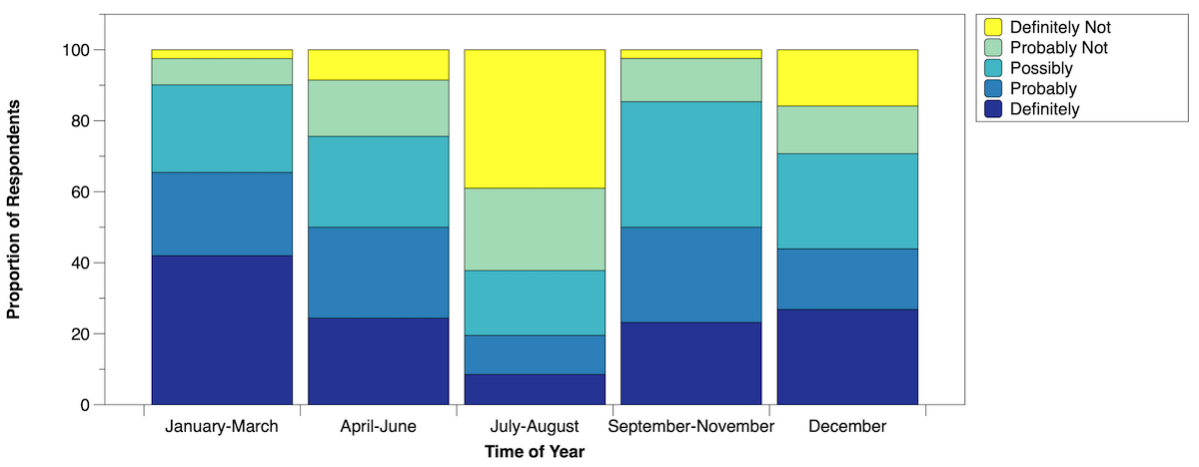
Likelihood of staff willing to work a weekend schedule at different times of the year.

Respondents were more willing to work extra weekends during nonsummer months, with a preference for the winter months (Figure 2).

#### Continued Participation in the Weekend Surgery Program

Of 82 staff members, 72 (88%) would be willing to continue working weekend lists and 56 (68%) would be willing to work weekends if cases were longer or more complex.

### Multivariable Analyses

**Figure 3:**
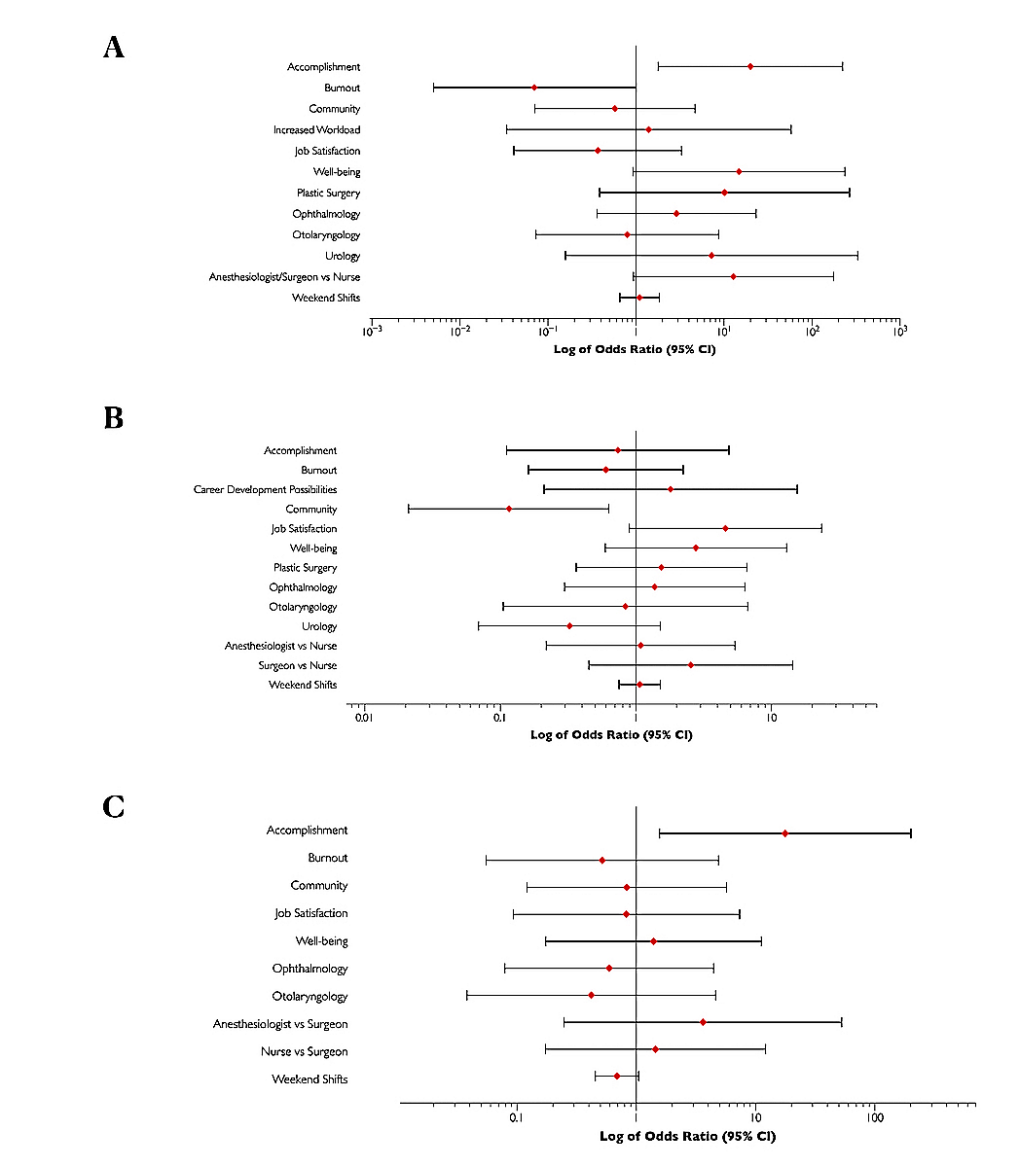
Multivariable logistic regression of factors associated with (A) satisfaction working weekend surgery; (B) willingness to work weekend lists with longer, more complex cases; and (C) willingness to work future weekend lists.

Participants’ sense of accomplishment was significantly associated with satisfaction with working on the weekend (OR 19.97, 95% CI 1.79-222.63; *P*=.02) (Figure 3A).

Figure 3B shows the factors associated with the willingness to work lists with more complex cases. A sense of community was associated with a willingness to participate in future weekend lists with more complex cases (OR 0.12, 95% CI 0.02-0.63; *P*=.01). A sense of accomplishment was associated with a willingness to participate in future weekend elective lists (OR 17.74, 95% CI 1.50-200.70; *P*=.02) (Figure 3C).

## Discussion

### Principal Findings

Our survey results demonstrate high overall satisfaction and positive feedback from staff working weekend elective surgical lists in the ORRACLE-Xtra program. A sense of accomplishment and community was associated with the intent to work weekend elective lists in the future, with a preference for nonsummer periods. Most staff would also be willing to work weekends that included longer and more complex cases.

### Comparison With Prior Work

The burden of COVID-19 on health care workers has been unprecedented, with several studies reporting health workers experiencing inadequate preparedness, emotional challenges, insufficient equipment and information, and work burnout [7,28-37]. Health care workers dealing directly with patients infected with COVID-19 in critical care areas experienced significant burden from increased workloads and the likelihood of dissatisfaction and burnout [33]. In contrast, as the pandemic led to a slowdown in surgical cases, surgical staff had relatively lower workloads. We piloted the ORRACLE-Xtra program during a period when the number of COVID-19 cases affecting the pediatric population was relatively low, during the winter months, and when several public lockdowns were in place. This situation may have contributed to our success in attracting volunteer staff to work the weekend lists.

Further, with ongoing cycles of pandemic lockdowns, working the weekends was associated with satisfaction and a sense of accomplishment among respondents. This finding is unexpected, especially during a pandemic, but may be explained by the opportunity offered to engage in meaningful work and contribute to reducing the growing surgical wait-list. Surgeons were grateful to secure extra operating time when regular weekday opportunities were reduced. The cancelation of surgeries and reduced weekday workload around the study period may also have mitigated the sense of work burnout from working on weekends. An in-depth assessment of burnout was beyond the scope of this study and may yield different results. However, as staff could choose their availability for weekend lists, this sense of autonomy may have mitigated feelings of loss of control, creating a positive experience and environment [38-40]. A few participants in the nursing group did report dissatisfaction, and work is underway to investigate causes using qualitative methodology.

Participants reported interest in working lists with longer and more complicated cases. This finding was surprising as the goal of the weekend list was low-acuity, high-volume cases to help minimize the number of required staff and stress levels among staff while managing cohorts of children who had their procedures deferred for more urgent cases during the waves of surgical slowdown. However, the sense of community associated with a willingness to work longer cases is consistent with reported efforts to improve workplace morale and reduce burnout by building cohesive teams [38-40].

### Impact on Future Planning

A key strength of our study is the high response rate and the availability of information for the planning of future weekend surgeries. Staff indicated their willingness to continue working weekend surgery, with preference for the nonsummer months of the year. This allowed institutional leaders to extend the ORRACLE-Xtra program for an additional 9 months, developing a weekend schedule cognizant of staff preferences and availability that addressed the surgical backlog while mitigating work-related burnout.

### Limitations

Our study has several limitations. First, as a survey, the data are limited to the questions posed to the respondents. However, we also included open-ended questions to allow respondents to provide additional feedback. The qualitative data helped further explain some of the unexpected findings from our study. Second, we used a single question to gather information on the sense of burnout, which may underestimate the scope of burnout among staff. While the sense of accomplishment and community associated with working weekend surgery are encouraging, it would be important to check in with staff regularly if the program continues. Finally, our results are based on a 3-month pilot and the status, needs, and demands will change as the pandemic unravels. We would need to conduct a follow-up survey to assess whether working weekend lists would lead to fatigue in the longer term.

### Conclusion

Staff working the first 3 months of the ORRACLE-Xtra program reported satisfaction with working weekends. A sense of accomplishment and community was associated with satisfaction and willingness to continue working weekend surgery, including longer, more complex cases. Integrating considerations of staff well-being and preferences is important for the implementation and planning of future surgical backlog recovery work. Institutions planning on implementing COVID-19 surgical backlog work would benefit from gathering key information from their staff.

## Appendix

Multimedia Appendix 1 Staff satisfaction survey.

Multimedia Appendix 2 Variables used in univariate and multivariate analyses.

Multimedia Appendix 3 Satisfaction with working weekend elective shifts.

## Abbreviations

CROSS: Consensus-Based Checklist for Reporting of Survey Studies
OR: odds ratio
ORRACLE-Xtra: Operating Room Ramp-Up After COVID-19 Lockdown Ends-Extra Lists

## Data Availability

The data sets generated and analyzed during this study are available from the corresponding author upon reasonable request.
