# Supplementary material for: Experiences of Health Care Professionals Working Extra Weekends to Reduce COVID-19–Related Surgical Backlog: Cross-sectional Study
Source: JMIR Perioper Med. 2022 Dec 6;5(1):e40209. doi: 10.2196/40209 (PMC9746672; doi:10.2196/40209)
Supplement: Multimedia Appendix 1 [file periop_v5i1e40209_app1.pdf]

Weekend Surgery Staff Satisfaction Survey - ORRACLE XTRA

Dear Colleague,

The weekend surgery is a new initiative at The Hospital for Sick Children. We would like to learn about your experience so we can understand how we did and how we can improve. These results will help inform future direction(s) for POS and the Hospital. Your responses will be anonymous.

Thank you for completing this survey.

The ORRACLE XTRA (Weekend Surgery) Steering Committee

The Hospital for Sick Children

Approved by Risk Management as a QI project

Demographics

Role:

◯ Anesthesiologist
◯ MDRD
◯ Nurse
◯ Patient Flow Coordinator
◯ Patient Information Clerk
◯ POCU Attendant
◯ Surgeon
◯ Trainee
◯ Other

Specify role:

____

FTE:

____

Area (check all that apply):

☐ Dentistry
☐ Ophthalmology
☐ Orthopaedics
☐ Otolaryngology
☐ Pre-Anesthesia Clinic
☐ Peri-Anesthesia Nursing
☐ Intra-Operative Nursing
☐ Plastic Surgery
☐ Registration
☐ Urology

How many weekend shifts have you worked?

◯ 1
◯ 2
◯ 3
◯ 4
◯ 5
◯ 6
◯ 7
◯ 8
◯ 9
◯ 10
◯ 11
◯ 12

Weekend Surgery Experience

How satisfied were you with working on the weekend elective list?

◯ Very Satisfied
◯ Satisfied
◯ Neither Satisfied nor Dissatisfied
◯ Dissatisfied
◯ Very Dissatisfied

To what extent did the weekend surgeries contribute to your sense of:

	Not at all	Very little	Somewhat	Quite a bit	A great deal
Accomplishment	◯	◯	◯	◯	◯
Burnout	◯	◯	◯	◯	◯
Career Development Possibilities	◯	◯	◯	◯	◯
Community	◯	◯	◯	◯	◯
Increased Workload	◯	◯	◯	◯	◯
Job Satisfaction	◯	◯	◯	◯	◯
Well-being	◯	◯	◯	◯	◯

What aspects of the weekend workflow, culture, patients, and operating room set-up contributed positively to your experience?

What aspects of the weekend workflow, culture, patients, and operating room set-up contributed negatively to your experience?

Weekend Surgery in the Future

I would be willing to participate in weekend surgery in the future:

◯ Definitely
◯ Probably
◯ Possibly
◯ Probably Not
◯ Definitely Not

Which weekend schedule(s) would you most likely consider working? (Check all that apply)

	Definitely	Probably	Possibly	Probably Not	Definitely Not
January-March	☐	☐	☐	☐	☐
April-June	☐	☐	☐	☐	☐
July-August	☐	☐	☐	☐	☐
September-November	☐	☐	☐	☐	☐
December	☐	☐	☐	☐	☐

Would you consider signing up for weekend surgery if the operative cases included longer (2-3 h) procedures or higher complexity procedures requiring inpatient admission?

◯ Definitely
◯ Probably
◯ Possibly
◯ Probably Not
◯ Definitely Not

Do you have any additional feedback or comments on your experience with weekend surgery?
