# Supplementary material for: Experiences of Health Care Professionals Working Extra Weekends to Reduce COVID-19–Related Surgical Backlog: Cross-sectional Study
Source: JMIR Perioper Med. 2022 Dec 6;5(1):e40209. doi: 10.2196/40209 (PMC9746672; doi:10.2196/40209)
Supplement: Multimedia Appendix 2 [file periop_v5i1e40209_app2.docx]

Multimedia Appendix 2

Variables used in univariate and multivariate analyses

| Variable | Type |
| --- | --- |
| Accomplishment | Yes |
|  | No(ref) |
| Burnout | Yes |
|  | No(ref) |
| Career Development Possibilities | Yes |
|  | No(ref) |
| Community | Yes |
|  | No(ref) |
| Increase Workload | Yes |
|  | No(ref) |
| Job Satisfaction | Yes |
|  | No(ref) |
| Well-being | Yes |
|  | No(ref) |
| Area Plastic Surgery | Yes |
|  | No(ref) |
| Area Ophthalmology | Yes |
|  | No(ref) |
| Area Orthopaedics | Yes |
|  | No(ref) |
| Area Otolaryngology | Yes |
|  | No(ref) |
| Area Urology | Yes |
|  | No(ref) |
| Weekend Shifts | Continuous |
| Role | Anesthesiologist |
|  | Nurse/POCU Attendant/Trainee |
|  | Surgeon |
| Satisfaction with Working on Weekends^a^ | Definitely |
|  | Definitely Not |
| Participating in future Weekend Surgery^a^ | Definitely |
|  | Definitely Not |
| More Complex case^a^ | Definitely |
|  | Definitely Not |
|  |  |

a = Outcome variable
