# Supplementary material for: Experiences of Health Care Professionals Working Extra Weekends to Reduce COVID-19–Related Surgical Backlog: Cross-sectional Study
Source: JMIR Perioper Med. 2022 Dec 6;5(1):e40209. doi: 10.2196/40209 (PMC9746672; doi:10.2196/40209)
Supplement: Multimedia Appendix 3 [file periop_v5i1e40209_app3.docx]

| Role | Very Satisfied | Satisfied | Neither Satisfied nor Dissatisfied | Dissatisfied | Very Dissatisfied |
| --- | --- | --- | --- | --- | --- |
| Anesthesiologist^a^ | 14/26 (53.8) | 23.1 (6/26) | 19.2 (5/26) | - | - |
| Surgeon | 47.1 (8/17) | 47.1 (8/17) | 0.0 (1/17) | - | - |
| Nurse | 23.5 (8/34) | 47.1 (16/34) | 23.5 (8/34) | 5.9 (2/34) | - |
| Trainee/Fellow | 100.0 (4/4) | - | - | - | - |
| POCU Attendant | - | 100.0 (1/1) | - | - | - |

Satisfaction with working weekend elective shifts

a Missing response from one respondent

N=82
